# Phenotypic and Molecular Characterization of Pyomelanin-Producing *Acinetobacter baumannii* ST2_Pas_;ST1816/ST195_Oxf_ Causing the First European Nosocomial Outbreak

**DOI:** 10.3390/microorganisms13030493

**Published:** 2025-02-22

**Authors:** Alessandro Leonildi, Alfredo Rosellini, Giulia Gemignani, Giusy Tiseo, Marco Falcone, Cesira Giordano, Simona Barnini

**Affiliations:** 1 Microbiology Unit, Azienda Ospedaliero-Universitaria Pisana, 56126 Pisa, Italy; 2 Organization of Hospital Services Unit, Azienda Ospedaliero-Universitaria Pisana, 56126 Pisa, Italy; 3 Infectious Diseases Unit, Azienda Ospedaliero-Universitaria Pisana, 56126 Pisa, Italy

**Keywords:** pyomelanin, nosocomial pathogens, healthcare-associated infections, outbreaks

## Abstract

*Acinetobacter baumannii* is one of the most successful and feared nosocomial pathogens. *A. baumannii* is considered a global threat in the healthcare setting, mainly owing to its ability to acquire multidrug resistance phenotypes. The *A. baumannii* pathogenesis is guided by its environmental persistence, as well as the production of numerous virulence factors. In several bacteria, the production of pigments, such as melanin, has indeed been linked with virulence and pathogenicity. Melanin is a brownish pigment, rarely observed in *A. baumannii*, that potentially reduces the susceptibility of the bacteria to host defense mechanisms and environmental insults. This study reports the first outbreak in Europe by pyomelanin-producing *A. baumannii* strains, in a tertiary-care university hospital in Pisa, Italy. Phenotypic and molecular analyses were performed.

## 1. Introduction

*Acinetobacter baumannii* is one of the most successful and feared nosocomial pathogens. It has gained major attention due to its capability to cause ventilator-associated as well as bloodstream infections in critically ill patients, its extensive antimicrobial resistance patterns, and its persistence in the healthcare environment. *A. baumannii* is considered a global threat in the healthcare setting, mainly owing to its ability to rapidly acquire multi-, extensive-, and even pan-drug-resistant phenotypes [1,2]. Antibiotic resistance has increased during the evolution of this microorganism, mainly because of the acquisition of mobile genetic elements such as transposons, plasmids, and integrons. Much of the spreading of *A. baumannii* can be directly attributed to the plasticity of its genome, which rapidly mutates when faced with adversity and stress. The Italian Nosocomial Infections Surveillance in Intensive Care Units (ICUs) network (SPINUTI) showed that *A. baumannii* was the most frequently reported microorganism (16.9%) as the cause of healthcare-associated infections in Italian ICUs during 2010–2011. Also, *A. baumannii* was responsible for 15% of sepsis in Italian ICUs from 2008 to 2017 [3]. In addition, the majority of isolates are considered to be multidrug-resistant (MDR) [4]. The mortality rate for ventilator-associated pneumonia caused by *A. baumannii* varies from 40 to 70% [5], while the mortality rate for bloodstream infections ranges from 28 to 43% [6]. For this reason, the World Health Organization (WHO) has included carbapenem-resistant *A. baumannii* (CRAB) in the critical group of bacteria that pose the greatest threat to human health, prioritizing research and development efforts for new antimicrobial treatments [7]. Over the past decade, the complex mechanisms that led to the emergence of *A. baumannii* as a feared human pathogen have been unraveled, particularly beyond the canonical drug resistance mechanisms that have been extensively studied in the past [8]. Some authors coined an expression that, in our opinion, describes perfectly the behavior of this microorganism: a ‘persist and resist’ strategy [9]. The *A. baumannii* pathogenesis is indeed guided by its environmental persistence, including resistance mechanisms to disinfection, desiccation, and oxidative stress; biofilm formation and maintenance; motility, in contrast to its name (‘*Acinetobacter*’ paradoxically stands for non-motile rod); and the production of numerous virulence factors, such as secretion systems, porins, surface glycoconjugates, and micronutrient acquisition systems, phospholipases, outer membrane vesicles, and serum resistance proteins [10]. In several bacteria, the production of pigments, such as melanin, have been linked with virulence and pathogenicity. Melanin is a brownish substance that potentially reduces the susceptibility of the microbe to host defense mechanisms and environmental insults. Depending on the pathway of synthesis, melanin may be given a different designation, in particular, the term pyomelanin was proposed for the brown pigment produced from tyrosine or phenylalanine through the accumulation of homogentisic acid [11]. Melanin production is a quite rare phenotype in *A. baumannii,* only three research groups have described this phenomenon [12,13,14]. Moreover, the mechanisms leading to melanin production have not yet been completely unveiled. In some bacteria, and most probably in *A. baumannii*, this pigment provides protection against oxidative stress and contributes to invasiveness and persistence. Pyomelanin enhances bacterial surface attachment, biofilm formation, extracellular electron transfer, resistance to heavy metals, iron reduction/acquisition, and induces the expression of virulence factors, which increase the adaptive response to environmental stress [15,16]. This study reports the occurrence of *A. baumannii* strains producing a brown diffusible pigment, visible on Mueller–Hinton agar cultures, which caused an outbreak in a tertiary-care university hospital in Pisa, Italy. Based on whole genome analyses of nine representative strains, we investigated the molecular bases involved in the production of this pigment and the relatedness of pyomelanin-producing *A. baumannii* (AbauPio+) with autochthonous pyomelanin-non producing *A. baumannii* strains (AbauPio-). Such an extensive outbreak caused by AbauPio+ has not, to our knowledge, been previously recorded elsewhere. We further investigated the molecular mechanisms beneath pyomelanin production and speculate a pathway involved in a defect in the catabolism of aromatic amino acids. The pyomelanin biosynthetic pathway is well known in *Pseudomonas* species: *Pseudomonas putida* metabolizesphenylalanine (Phe) and tyrosine (Tyr) through a peripheral pathway, involving the hydroxylation of Phe to Tyr by a phenylalanine hydroxylase (PhhAB), the conversion of Tyr into 4-hydroxyphenylpyruvate by a tyrosine aminotransferase (TyrB), and the formation of homogentisic acid (HGA) by a 4-hydroxyphenylpyruvate dioxygenase (Hpd) as the central intermediate. HGA is then catabolized by a central catabolic pathway that involves the homogentisate dioxygenase (HmgA), fumarylacetoacetase (HmgB), and maleylacetoacetate isomerase (HmgC), finally yielding fumarate and acetoacetate [17]. So far, mutations or deletions that result in the loss of HmgA function have been described, as well as the overexpression of *hmgR*, that leads to an accumulation of HGA [18]. The accumulated HGA is then secreted from the cell via the homogentisic acid transport proteins (HatABCDE ABC transporter), where it auto-oxidizes, and self-polymerizes to form pyomelanin [19] (Supplementary Data S3). Thus, we performed an in silico comparative genomic analysis to identify and characterize the genes involved with pyomelanin production and we revealed a defect in the catabolic pathway of fumarate biosynthesis, which leads to the production of pyomelanin via the accumulation of HGA.

## 2. Methods

### 2.1. Epidemiological Context

The Azienda Ospedaliero-Universitaria Pisana is a tertiary-care University Hospital, where almost 50,000 patients are hospitalized every year. There are General wards, Cardiology, Endoscopy, Hematology, Gynecology, Paediatrics, a Neonanal Unit, a Maternity Ward, Surgeries, a Burn Unit, an Emergency department, and nine Intensive Care Units. There is an intense surveillance system for monitoring MDR microorganisms (particularly carbapenemase-producing *Enterobacterales*, *A. baumannii complex,* and *Stenotrophomonas maltophilia*), which consists of a rectal swab analysis (molecular and/or culture) at the admission and, depending on the ward, a weekly or bi-weekly rectal swab analysis during the hospitalization period. On the positive result, an “alert” event is generated by the OpenLIS software v21.1.0 (Engineering Software Laboratory, Belgrade, Serbia, https://www.engserbia.com/) and automatic emails are sent to the ward where the patient is hospitalized, to Microbiology, Infectivology, Infection Control employees, and Medical Direction. Epidemiological data regarding the *A. baumannii* isolation site, ward, and antimicrobial resistance pattern were extracted using the appropriate query on the OpenLIS software from January 2018 to December 2023.

### 2.2. Phenotypic and Molecular Characterization of Strains

A total of forty clinical *A. baumannii* strains, dark in color, on Muller–Hinton agar were isolated from unique patients during May–December 2023. Various biological samples were cultured on common isolation media in routine diagnostics and incubated at 37 °C overnight. Colonies suspected to be AbauPio+ were identified by matrix-assisted laser desorption/ionization time-of-flight mass spectrometry (MALDI-TOFMS) (Bruker Daltonics GmbH, Bremen, Germany) and cultured on Muller–Hinton agar (Thermo Fisher Scientific, Waltham, MA, USA, https://www.thermofisher.com/). Antimicrobial susceptibility testing was performed by broth microdilution assay panels (Micronaut, Merlin, Germany, https://www.merlin-diagnostika.de/) using the Pickolo—Freedom EVO system (TECAN, Männedorf, Switzerland, https://www.tecan.com/). Cefiderocol inhibition halos were assessed by the disk-diffusion method, according to the manufacturer’s instructions (Thermo Fisher Scientific, https://www.thermofisher.com/). Minimum inhibitory concentrations (MICs) were interpreted according to the latest European Committee on Antimicrobial Susceptibility Testing (EUCAST) guidelines. The strains were frozen in rich broth with 10% glycerol added for further investigation. The strains’ behavior was evaluated under the exposure to different temperatures of incubation: 25 °C, 30 °C, and 37 °C. The extracellular production of melanin was evaluated by extracting the pigment from cultured AbauPio + agar plates (modified from Loi 2020 [20]). Briefly, small portions of pigmented agar were dissolved in HCl 6 M and incubated for 2 h at room temperature, then, the pellet was washed twice in MilliQ water and resuspended in NaOH 0.5 M. This solution obtained was checked by a spectrophotometer at 200–600 nm to determine peak absorbance. In addition, two couples of AbauPio+ (M-P, F-P) and AbauPio-(M-NP, F-NP) were challenged with hydrogen peroxide (H_2_O_2_) agar-diffusion and broth microdilution tests. For the H_2_O_2_ agar-diffusion test, 50 µL of hydrogen peroxide was spotted at scalar dilution, from 0.75% to 0.08%, on Mueller–Hinton agar plates previously inoculated with 0.5 McF bacterial suspension. After an overnight incubation at 37 °C, diameters of growth inhibition halos were measured; 50 µL of saline was tested as a negative control. For the H_2_O_2_ broth microdilution test, hydrogen peroxide was diluted in Mueller–Hinton broth from 1.5% to 1.46 × 10 ^−6^%. The bacterial suspensions were standardized at 0.5 McF in sterile and then 50 µL was added to 11 mL of Mueller–Hinton broth to obtain a standard inoculum of 5 × 10^5^ CFU/mL. Then, 100 µL of the bacterial suspension was distributed in the wells with the scalar dilution of hydrogen peroxide. After an incubation for 18–20 h at 35 °C, the hydrogen peroxide concentration able to inhibit bacterial growth was identified. Moreover, every well without visible bacterial growth was cultured on blood agar plates to verify the bactericidal activity. Nine strains underwent genomic analyses. Total DNA was extracted from fresh cultures using the QIAamp DNA Mini Kit (Qiagen, https://www.qiagen.com/) according to the manufacturer’s instructions. The concentration and purity of the extracted DNA were determined with a Qubit^®^ 2.0 fluorometer using the dsDNA BR Assay Kit (Life Technologies, Carlsbad, CA, USA). A DNA library was prepared using the Nextera XT DNA Library Preparation Kit (Illumina Inc., San Diego, CA, USA) according to manufacturer’s instructions, and was then run on a MiSeq system (Illumina Inc.) to generate 250 bp paired-end reads. Then, a multivariable Cox regression analysis was performed.

### 2.3. Software and Tools Used for Genomic Analysis

De novo assembly was performed by SPAdes Genome Assembler tool [21] using UGENE v33 [22], a free open-source software for DNA and protein sequence visualization, alignment, assembly, and annotation, after quality trimming (Qs  ≥  28). Assembled genomes were uploaded to the web tools ResFinder (http://genepi.food.dtu.dk/resfinder) [23] to identify acquired resistance genes as well as to MobileElementFinder (https://pypi.org/project/MobileElementFinder/) [24] to detect mobile genetic elements. Then, the sequences were uploaded on RAST (Rapid Annotation using Subsystem Technology, https://rast.nmpdr.org/), a fully-automated web service for annotating bacterial genomes [25]. RAST was also used for the in silico metabolic reconstruction of pyomelanin-producing *A. baumannii* and for comparisons both function-based and sequence-based with other *A. baumannii* genomes. The sequences are available from the NCBI Archive under the Bioproject names PRJNA1007229, PRJNA926509, PRJNA1102832, and PRJNA1102841; accession numbers are reported in Table 1. The DNA sequences of *hmgA* (NC_016603.1:3098928-3099560), hmgB (NC_016603.1:3097634-3098941), *hmgR* (NZ_AP014630.1:c3200330-3199053), and *hmgC* (NM_001046668.1) were used as references for detecting possible gene mutations associated with pyomelanin production. Insertion sequence (IS) elements were identified using the web tool ISfinder (https://isfinder.biotoul.fr/) [26]. The ISAba125 reference sequence (AY751533) was BLASTed (https://blast.ncbi.nlm.nih.gov/) against the whole genome sequence of all the isolates. Manual sequence alignment was performed by MEGA (Molecular Evolutionary Genetics Analysis) software (https://www.megasoftware.net/) [27]. The chewBBACA software v3.3.10 was run in Conda environment for the creation, evaluation, and use of core genome (cg) multilocus sequence typing (MLST) schemas, based on an ad hoc structure including 2390 alleles [28]. The ChewTree tool was used to calculate a phylogenetic tree from chewBBACA alleles on the web tool ARIES (Advanced Research Infrastructure for Experimentation in Genomics—Galaxy Instance at ISS, https://irida.iss.it/irida-phantastic/login) [29]. The tree was visualized by PHYLOViZ (https://www.phyloviz.net/). In addition, 17 genomes of AbauPio- isolated from April 2020 to November 2022 were used as comparators (PRJNA1007229, PRJNA926509).

## 3. Results

### 3.1. Phenotypic and Epidemiologic Analysis

**Figure 1:**
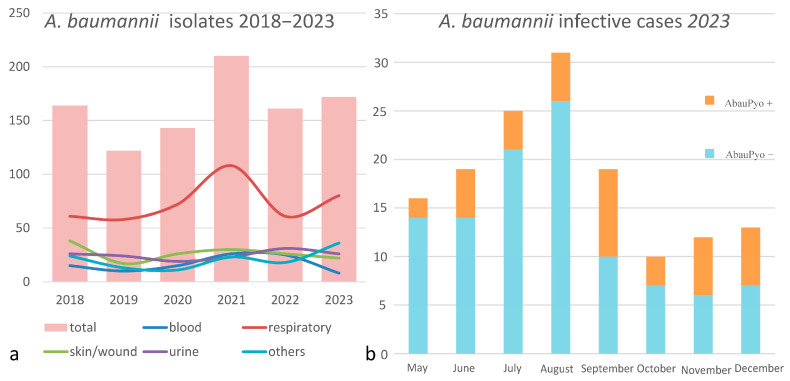
(**a**) The proportion of *A. baumannii* isolation during a 5-year period (2018–2023); the different colored lines represent different sites of isolation. (**b**) *A. baunannii* infection cases from May to December 2023, distinguished by the production of the pigment.

**Figure 2:**
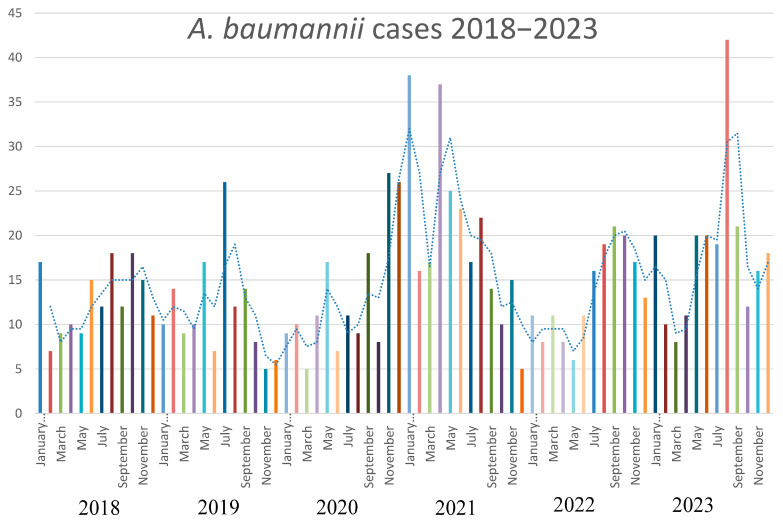
The epidemiology of *A. baumannii* during a 5-year period (2018–2023).

The strains of *A. baumannii* were correctly identified at the species level by MALDI-TOF MS, with score values above 2.3. Epidemiological data and strains features are reported in Table 1. In relation to their antimicrobial susceptibility pattern, performed by broth microdilution assay, all the strains were shown to be multi-drug-resistant. Regarding the hospital epidemiology, the proportion of *A. baumannii* isolation was reported in Figure 1a. Figure 1b reports the isolates from infections from May to December 2023, divided by the production of the pigment. The majority of strains were isolated from respiratory samples with an increasing isolation in 2021, both for respiratory samples and for blood cultures. Regarding the susceptibility pattern, the proportion of amikacin-resistant strains went from 67% in 2018 to 90% in 2023, meropenem-resistant strains went from 80% in 2018 to 91% in 2023, while there is an inversion in the proportion of colistin-resistant strains, which went from 4% in 2018 to 1% in 2023. In Figure 2, we reported the epidemiology of all of the *A. baumannii* isolation from 2018 to 2023, divided by months. As shown, the prevalence is higher in warmer months, except during the COVID-19 pandemic, when all the infection control measures were disrupted. The production of pyomelanin was more evident at 30 °C and 37 °C rather than 25 °C (Supplementary Data S1). In the H_2_O_2_ agar-diffusion test, the AbauPio+ strains showed growth at 0.08% of hydrogen peroxide, whereas, at the same hydrogen peroxide concentration, 24 mm of growth inhibition halos were detected for the AbauPio- strains. Differential growth inhibition halos for AbauPio+ and AbauPio- strains are listed in Table 2 (image in Supplementary Data S2). The minimal H_2_O_2_ concentration that inhibited the growth of bacteria, evaluated by the H_2_O_2_ broth microdilution test, was 0.04% for AbauPio+ strains and 0.00015% for AbauPio-. The bacteriostatic hydrogen peroxide concentrations were also bactericidal. After the extraction, the brown pigment was analyzed by a spectrophotometer and a peak absorption was observed at 215 nm in the UV region, the specific wavelength of melanin [20].

### 3.2. Molecular Analysis

**Figure 3:**
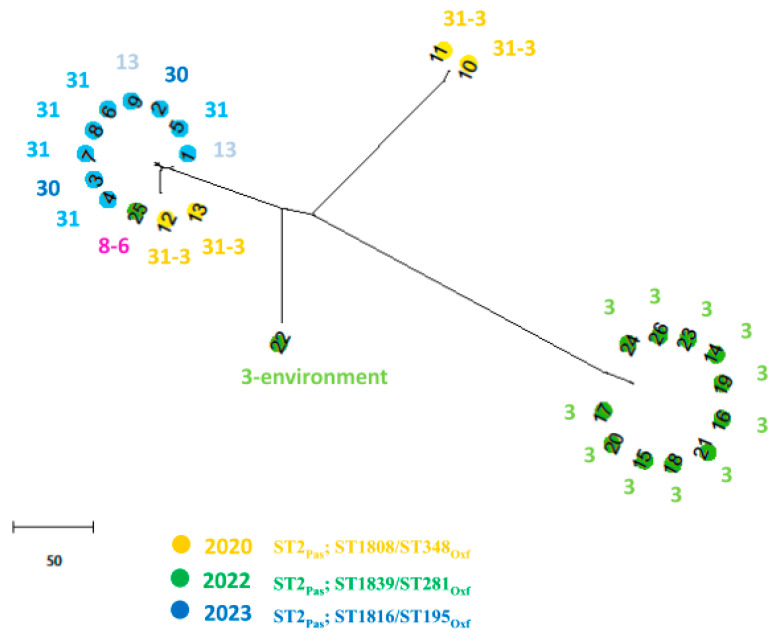
A phylogenetic tree based on the allelic mismatch between 23 *A. baumannii* genomes. The chewBBACA software was run in the Conda environment for the creation, evaluation, and use of core genome (cg) multilocus sequence typing (MLST) schemas, based on an ad hoc structure including 2390 alleles. Different colors represent different years of isolation and different sequence types (according to the Oxford schema). Numbers inside the colored dots represent the number of the genome, while the number outside the colored dots represents the number of the ward where the strain was isolated.

Resistome prediction analyses unveiled different combinations of β-lactamases, including OXA-23, OXA-25, OXA-66, TEM-1D, and ADC-25 enzymes. Several aminoglycoside resistance genes, including *aph(6)-Id, aph(3′)-Ia, aph(3″)-Ib, aadA1, armA,* and the *aac(6′)-Ib-cr* gene involved also in fluoroquinolone-resistance, were also detected and are reported in Table 1. In AbauPio- cefiderocol-resistant strains, a missense mutation in PBP3 was detected. Those isolates also harbored a mutation in the TonB-dependent siderophore receptor gene *piuA,* that showed a frameshift mutation causing a premature stop codon (K384fs). Moreover, the *fepA* gene, which is the orthologous to *pirA*, was interrupted by a transposon insertion P635-IS*Aba125* (IS*30* family), and was associated with the resistance to cefiderocol [30]. The core genome whole genome sequencing analysis revealed that the isolate belonged to sequence type 2 (ST) International Clone II, according to the MLST Pasteur database, and to ST1816/ST195, according to the Oxford database (Table 1). The nine AbauPio+, together with seventeen AbauPio- strains, were selected for wgMLST comparison. Figure 3 shows a phylogenetic tree based on the allelic mismatch between these genomes. The different Oxford MLST types are highlighted by different colors: three clonally related clusters of isolates are observed, and in agreement with the Oxford schema classification, except for one sample (number 25, which clustered in an unconventional way). Strain number 22, isolated from the hospital environment, was considered the outgroup. The three clusters are separated according to the timing of isolation (2020, 2022, 2023) and to nosocomial setting, since different wards are involved and the Oxford schema is able to highlight those aspects. IS*Aba125* is a transposable insertion sequence of 1026nucleotides, a member of the widely distributed IS*30* family. In strains number six and seven, the IS*Aba125* was found in 128 nucleotides upstream of the fumarylacetoacetase gene, in a palindromic region rich in A and T nucleotides resembling a regulatory region, most probably causing a rearrangement of the pyomelanin genes cluster and a misidentification of the site by the RNA polymerase, leading to an accumulation of HGA, which is then secreted from the cell via the HatABCDE ABC transporter, where it auto-oxidizes and self-polymerizes to form pyomelanin. In the remaining AbauPyo+ strains, IS*Aba125* was found in isolated contigs and the reconstruction of the frame was not possible.

## 4. Discussion

Carbapenem-resistant *Acinetobacter baumannii* represents one of the pathogens with a higher mortality rate in nosocomial settings [31]. The *A. baumannii* outbreaks have been associated with high-risk pandemic lineages, named International Clones (ICs), characterized by a high capacity to persist in clinical environments and by presenting a broad antimicrobial resistance profile [32]. The exposure of *A. baumannii* to the selective pressure of potent antimicrobials, primarily in the ICUs, has gradually led to a global prevalence of *A. baumannii* strains that are resistant to all β-lactams, including carbapenems. Outbreaks caused by such strains have been identified in several ICUs worldwide and, in many instances, have been associated with strains that are resistant to all available antibacterial agents. Also, in our hospital epidemiology, the proportion of carbapenem-resistant strains is widely distributed, reaching 90% of all the isolations, and strategies for new antimicrobial approaches were attempted [33,34]. Interestingly, an inversion in colistin resistance was registered from 2018 to 2023, most probably because, in our hospital, the use of this antibiotic had decreased in that period, diminishing the selective pressure on the bacteria. Considering the epidemiology from 2018 to 2023, we noticed that the proportion of isolation is higher in warmer months, except during the COVID-19 pandemic, most probably because of the lack of infection control measures. The majority of *A. baumannii* strains were cultures from respiratory samples with an increase during the COVID-19 pandemic. The same trend was registered also for blood cultures. This may be explained considering the rise in patients admitted to ICUs and subjected to assisted-control ventilation during the COVID-19 pandemic, but also the lack of infection control measures registered in that period. Beyond its environmental persistence and its aptitude to accumulate a large variety of resistance mechanisms, the ability to generate biofilm, the production of a capsule, the presence of lipopolysaccharide and outer-membrane proteins, and the secretion of hydrolytic enzymes have been highlighted as virulence factors of *A. baumannii* [35]. Melanin is a brownish pigment that has also been linked with the virulence and pathogenicity of microbes able to produce this substance [36]. However, the pigmentation of clinical strains of *A. baumannii* is quite a rare phenotype [12,13,14] and such outbreaks have not yet been described elsewhere. The overproduction of pyomelanin in *A. baumannii* has been described as occurring due to changes in the tyrosine metabolic pathway linked to the deletion of the *hmgA* by IS*Aba1* [14]. From May to December 2023, the production of a brown diffusible pigment on Muller–Hinton agar was observed in forty isolates identified as *A. baumannii,* obtained from patients hospitalized in a tertiary-care hospital in Pisa, Italy. The outbreak started in one of the ICUs and overran in several other wards. It was verified that the production of pigment at higher temperatures was evident, as reported by Fonseca at al. (2020) [12], due to the induction of *melA* (*hpd*), which is responsible for HGA synthesis, suggesting the role of this biosynthetic pathway in the pyomelanin production in Abaupio+ strains. To explore the molecular basis of pigment production, we investigated the key genes in the pathway of pigment production, focusing right on HGA biosynthesis. The results revealed that IS*Aba125*, an insertion sequence belonging to the IS30 family, inserts upstream of the fumarylacetoacetase gene, possibly resulting in a steric hindrance in the regulatory/promoter region, with consequences of the impossibility of *hmgB* transcription, the accumulation of HGA, and its conversion into pyomelanin [11]. The impact of pyomelanin on the phenotype of *A. baumannii* has been studied: Fonseca et al. [12] considered that there might be a connection between pyomelanin production and virulence; Zhao et al. [13] revealed that the pigment may offer a potential protection effect for bacteria under UV radiation. In addition, pyomelanin-producing *A. baumannii* can show resistance to a wide range of antimicrobial drugs [14], which in turn could become a new challenge in the clinical setting, especially in the ICUs. In our experience, an intense resistance to oxidative stress was documented. More interestingly, during the period of AbauPio+ strains isolation, the hospital protocols of wards disinfection involved precisely hydrogen peroxide (Perox, Pestnet, diluted at 1% of H_2_O_2_). The study of *A. baumannii* genomics has provided an expanded view of the adaptation and virulence capacities of this tough bacterial species [37]. The IS*Aba125* insertion sequence is an expression of this plasticity, and the strains reported here are an emblematic example of their genetic variability. In Abaupio+ strains, IS*Aba125* inserts upstream of a gene involved in pyomelanin production; in Abaupio- strains, IS*Aba125* inserts within the *fepA* gene, causing resistance to cefiderocol [30]. The ability to interfere with gene expression has already been described for IS*Aba125*. This insertion sequence is able to insert upstream of the *bla*ADC gene in the promoter region, causing an overexpression of the gene [38], as well as inside the gene coding for the outer membrane protein CarO OMP, leading to a carbapenem-resistant profile [39]. In addition, IS*Aba125* has also been associated with gene variants of the *bla*NDM carbapenemase in the family *Enterobacterales* [40]. Insertion sequence elements, such asIS*Aba125*, could modulate antibiotic resistance depending on the antibiotic treatment [41], as well as virulence factors, such as the production of pyomelanin, as reported here. To our knowledge, this is the first report of a large outbreak caused by ST2_Pas_;ST1816/ST195_Oxf_ *A. baumannii* strains, where a biosynthetic pathway compatible with pyomelanin production was investigated. Further studies are required to understand which is the trigger for the production of pyomelanin and to establish the impact of pigment production on the pathogenesis of *A. baumannii*. In our multivariable Cox regression analysis, there was no statistically relevant difference in 30 days mortality rate between the AbauPio+ group (31.8%) and AbauPio- group (41.9%), also there were no statistically relevant differences between the two groups in the development of bacteremia. However, we prefer not to generalize this concept, since the groups analyzed were too small and therefore not adjusted for sex, age, and therapy; for this reason, we invite other clinicians to study this phenomenon to better clarify the clinical impact of AbauPio+ infections. Finally, due to the association of pyomelanin with virulence in bacteria, the surveillance of pigment production by clinical *A. baumannii* strains should be implemented and monitored as part of the hospital surveillance programs to contain the dissemination of these strains.

## Figures and Tables

**Table 1:** *A. baumannii* strains: epidemiological, genotypical, and phenotypical features. M and F are the strains’ names, P stands for pyomelanin-producers; NP stands for non pyomelanin-producers.

		HYDROGEN PEROXIDE %			SALINE
	0.75%	0.37%	0.16%	0.08%	CTRL
**MP**	34	24	10	0	0
**MNP**	40	32	30	24	0
**FP**	36	25	10	0	0
**FNP**	36	32	30	24	0

**Table 2:** Hydrogen peroxide agar-diffusion test: two couples of pigmented (M-P, F-P) and non-pigmented (M-NP, F-NP) *A. baumannii* strains from the same patients (M and P: each of them had a pigmented and a non-pigmented strain) were tested. Growth inhibition halos for pigmented (MP, FP) and non-pigmented (MNP, FNP) *A. baumannii* strains are reported (diameters in mm). ✓ stands for “present”.

Strain	Isolation Date (Source)	Breakpoints				Genome Size (base)	Sequence Type	Resistome		Mobilome	Virulence					Accession Number
		Amikacin (mg/L)	Meropenem (mg/L)	Colistin (mg/L)	Cefiderocol (mm)		MLST	Aminoglycoside Resistance	B-Lactam Resistance	MGEs	Colicin V	RecA	Pili Type	Porin	Pyomelanin	
1	28/05/23 (sputum)	>16	64	≤0.5	21 mm	3,987,231	ST2_Pas_; ST1816/ST195_Oxf_	aph(6)-Id, aph(3′)-Ia, aph(3″)-Ib, armA	OXA -23, OXA-66, TEM-1D, and ADC-25	ISAba24, ISAba26, IS17, is26, ISAba125	✓	✓	I/II/IV	OprB, Aquaporin Z	✓	SAMN41030183
2	14/06/23 (urine)	>16	32	≤1	22 mm	3,999,467	ST2_Pas_; ST1816/ST195_Oxf_	aph(6)-Id, aph(3′)-Ia, aph(3″)-Ib, armA	OXA -23, OXA-66, TEM-1D, and ADC-25	ISAba24, ISVsa3, ISAba26, IS17, ISAba25	✓	✓	I/II/IV	OprB, Aquaporin Z	✓	SAMN41030184
3	20/06/23 (CVC swab)	>16	32	≤1	22 mm	3,995,867	ST2_Pas_; ST1816/ST195_Oxf_	aph(6)-Id, aph(3′)-Ia, aph(3″)-Ib, armA	OXA -23, OXA-66, TEM-1D, and ADC-25	ISAba24, ISVsa3, ISAba26, IS17, IS26, ISAba125	✓	✓	I/II/IV	OprB, Aquaporin Z	✓	SAMN41030185
4	23/06/23 (BAS)	>16	64	1	22 mm	3,998,140	ST2_Pas_; ST1816/ST195_Oxf_	aph(6)-Id, aph(3′)-Ia, aph(3″)-Ib, armA	OXA -23, OXA-66, TEM-1D, and ADC-25	ISAba24, ISVsa3, ISAba26, IS17, ISAba125	✓	✓	I/II/IV	OprB, Aquaporin Z	✓	SAMN41030186
5	27/06/23 (BAS)	>16	64	≤0.5	21 mm	3,990,297	ST2_Pas_; ST1816/ST195_Oxf_	aph(6)-Id, aph(3′)-Ia, aph(3″)-Ib, armA	OXA -23, OXA-66, TEM-1D, and ADC-25	ISAba24, ISVsa3, ISAba26, IS17, IS26, ISAba25	✓	✓	I/II/IV	OprB, Aquaporin Z	✓	SAMN41030187
6	27/06/23 (BAL)	>16	>64	≤0.5	22 mm	3,988,632	ST2_Pas_; ST1816/ST195_Oxf_	aph(6)-Id, aph(3′)-Ia, aph(3″)-Ib, armA	OXA -23, OXA-66, TEM-1D, and ADC-25	ISAba24, ISVsa3, ISAba26, IS17, IS26, ISAba125	✓	✓	I/II/IV	OprB, Aquaporin Z	✓	SAMN41030188
7	12/07/23 (BAL)	>16	>64	≤0.5	22 mm	3,989,774	ST2_Pas_; ST1816/ST195_Oxf_	aph(6)-Id, aph(3′)-Ia, aph(3″)-Ib, armA	OXA -23, OXA-66, TEM-1D, and ADC-25	ISAba24, ISVsa3, ISAba26, IS17, ISAba125	✓	✓	I/II/IV	OprB, Aquaporin Z	✓	SAMN41030189
8	13/07/23 (BAS)	>16	64	≤0.5	22 mm	3,988,236	ST2_Pas_; ST1816/ST195_Oxpiraginef_	aph(6)-Id, aph(3′)-Ia, aph(3″)-Ib, armA	OXA -23, OXA-66, TEM-1D, and ADC-25	ISAba24, ISVsa3, ISAba26, IS17, ISAba125	✓	✓	I/II/IV	OprB, Aquaporin Z	✓	SAMN41030190
9	13/07/23 (BAL)	>16	64	0.5	20 mm	3,990,333	ST2_Pas_; ST1816/ST195_Oxf_	aph(6)-Id, aph(3′)-Ia, aph(3″)-Ib, armA	OXA -23, OXA-66, TEM-1D, and ADC-25	ISAba24, ISVsa3, ISAba26, IS17, IS26, ISAba25	✓	✓	I/II/IV	OprB, Aquaporin Z	✓	SAMN41030191
10	24/04/20 (blood)	>16	>64	1	6 mm	3,846,537	ST2_Pas_; ST1808/ST348_Oxf_	aph(6)-Id, aph(3″)-Ib, armA	OXA -72, OXA-66, TEM-1D, and ADC-25	ISVsa3, ISAba26, ISAba13, ISEc28, ISEc29, ISAba1, IS17, IS26, ISAba36	✓	✓	I/IV	OmpA, OprD, OprB, Aquaporin Z	-	SAMN41030730
11	24/04/20 (blood)	>16	>64	1	20 mm	3,790,446	ST2_Pas_; ST1808/ST348_Oxf_	aph(6)-Id, aph(3″)-Ib, armA	OXA -72, OXA-66, TEM-1D, and ADC-25	ISVsa3, ISAba26, ISAba13, ISEc29, IS17, IS26, ISAba36	✓	✓	I/IV	OmpA, OprD, OprB, Aquaporin Z	-	SAMN41030731
12	24/04/20 (skin)	>16	64	≤0.5	6 mm	3,999,068	ST2_Pas_; ST1808/ST348_Oxf_	aph(3′)-Ia, aph(3″)-Ib, armA,	OXA -23, OXA-66, TEM-1D, and ADC-25	ISAba24, ISVsa3, ISAba125, ISAba26, IS17, IS26	✓	✓	I/IV	OmpA, OprD, OprB, Aquaporin Z	-	SAMN41030732
13	24/04/20 (skin)	>16	64	≤0.5	20 mm	4,032,758	ST2_Pas_; ST1808/ST348_Oxf_	aph(3′)-Ia, aph(3″)-Ib, armA,	OXA -23, OXA-66, TEM-1D, and ADC-25	ISAba24, ISVsa3, ISAba125, ISAba26, IS17, ISAba36, IS26	✓	✓	I/IV	OmpA, OprD, OprB, Aquaporin Z	-	SAMN41030733
14	03/11/22 (rectal swab)	>16	32	≤0.5	<17 mm	3,975,455	ST2_Pas_; ST1839/ST281_Oxf_	aph(3′)-Ia, aph(3″)-Ib, armA, aadA1, aac(6′)-Ib-cr	OXA -225, OXA-66, ADC-25	ISAba24, ISVsa3, ISAba125	✓	✓	I/IV	OmpA, OprD, OprB, Aquaporin Z	-	SAMN41030734
15	28/10/22 (skin)	>16	32	≤0.5	<17 mm	3,976,418	ST2_Pas_; ST1839/ST281_Oxf_	aph(3′)-Ia, aph(3″)-Ib, armA, aadA1, mazzettiaac(6′)-Ib-bonuccellicr	OXA -26, OXA-66, ADC-25	ISAba24, ISVsa3, ISAba125, IS26	✓	✓	I/IV	OmpA, OprD, OprB, Aquaporin Z	-	SAMN41030735
16	07/11/22 (blood)	>16	32	≤0.5	<17 mm	3,974,827	ST2_Pas_; ST1839/ST281_Oxf_	aph(3′)-Ia, petracciniaph(3″)-Ib, ardi garbomA, aadA1, aac(6′)-Ib-cr	OXA -225, OXA-66, OXA-23, ADC-25	ISAba24, ISVsa3, ISAba125	✓	✓	I/IV	OmpA, OprD, OprB, Aquaporin Z	-	SAMN41030736
17	27/10/22 (skin)	>16	16	≤0.5	12 mm	3,971,629	ST2_Pas_; ST1839/ST281_Oxf_	aph(3′)-Ia, aph(3″)-Ib, armA, aadA1, aac(6′)-Ib-cr	OXA-66, OXA-23, ADC-25	ISAba24, ISVsa3, ISAba125, IS26	✓	✓	I/IV	OmpA, OprD, OprB, Aquaporin Z	-	SAMN41030737
18	01/11/22 (CVC)	>16	32	≤0.5	11 mm	3,972,425	ST2_Pas_; ST1839/ST281_Oxf_	aph(3′)-Ia, aph(3″)-Ib, armA, aadA1, aac(6′)-Ib-cr	OXA-66, OXA-23, ADC-25	ISAba24, ISVsa3, ISAba125	✓	✓	I/IV	OmpA, OprD, OprB, Aquaporin Z	-	SUB12614636
19	07/11/22 (blood)	>16	16	≤0.5	<17 mm	3,974,920	ST2_Pas_; ST1839/ST281_Oxf_	aph(3′)-Ia, aph(3″)-Ib, armA, aadA1, aac(6′)-Ib-cr	OXA-66, OXA-23, ADC-25	ISAba24, ISVsa3, ISAba125, IS26	✓	✓	I/IV	OmpA, OprD, OprB, Aquaporin Z	-	SAMN41030738
20	10/10/2022 (skin)	>16	16	≤1	<17 mm	3,971,101	ST2_Pas_; ST1839/ST281_Oxf_	aph(3′)-Ia, aph(3″)-Ib, armA, aadA1, aac(6′)-Ib-cr	OXA -225, OXA-66, ADC-25	ISAba24, ISVsa3, ISAba125, IS26	✓	✓	I/IV	OmpA, OprD, OprB, Aquaporin Z	-	SAMN41030739
21	06/10/2022 (blood)	>16	16	≤1	<17 mm	4,121,556	ST2_Pas_; ST1839/ST281_Oxf_	aph(3′)-Ia, aph(3″)-Ib, armA, aadA1, aac(6′)-Ib-cr	OXA -225, OXA-66, ADC-25, blaCMY-33, blaOXA-10	IS26, ISEc9, ISAba24, Tn6196, ISVsa3, IS5075, ISAba125	✓	✓	I/IV	OmpA, OprD, OprB, Aquaporin Z	-	SAMN41030740
22	Environmental swab	>16	32	≤0.5	20 mm	3,942,955	ST2_Pas_; ST1839/ST281_Oxf_	aph(3′)-Ia, aph(3″)-Ib, armA, aadA1, aac(6′)-Ib-cr	OXA -23, OXA-66, ADC-25	Tn6207, ISEc29, ISAba26,IS26	✓	✓	I/IV	OmpA, OprD, OprB, Aquaporin Z	-	SAMN41030741
23	22/08/22 (BAL)	>16	16	≤0.5	18 mm	3,975,018	ST2_Pas_; ST1839/ST281_Oxf_	aph(3′)-Ia, aph(3″)-Ib, armA, aadA1, aac(6′)-Ib-cr	OXA -225, OXA-66, ADC-25	ISAba24, ISVsa3, ISAba125, IS26	✓	✓	I/IV	OmpA, OprD, OprB, Aquaporin Z	-	SAMN41030742
24	17/09/22 (blood)	>16	16	≤0.5	9 mm	3,977,422	ST2_Pas_; ST1839/ST281_Oxf_	aph(3′)-Ia, aph(3″)-Ib, armA, aadA1, aac(6′)-Ib-cr	OXA -225, OXA-66, ADC-25	ISAba24, ISVsa3, ISAba125	✓	✓	I/IV	OmpA, OprD, OprB, Aquaporin Z	-	SAMN41030743
25	14/06/22 (wound)	>16	32	≤0.5	9 mm	3,935,147	ST2_Pas_; ST1839/ST281_Oxf_	aph(3′)-Ia, aph(3″)-Ib, armA, aadA1, aac(6′)-Ib-cr	OXA -23, OXA-66, ADC-25, TEM-1D	ISVsa3, ISEc29, ISAba125, ISAba26, IS17, IS26	✓	✓	I/IV	OmpA, OprD, OprB, Aquaporin Z	-	SAMN41030744
26	15/09/22 (blood)	>16	16	≤0.5	14 mm	3,972,320	ST2_Pas_; ST1839/ST281_Oxf_	aph(3′)-Ia, aph(3″)-Ib, armA, aadA1, aac(6′)-Ib-cr	OXA -225, OXA-66,	ISAba24, ISVsa3, ISAba125	✓	✓	I/IV	OmpA, OprD, OprB, Aquaporin Z	-	SAMN41030745

## Data Availability

The original contributions presented in this study are included in the article/supplementary material. Further inquiries can be directed to the corresponding author.

## Supplementary Materials

The following supporting information can be downloaded at https://www.mdpi.com/article/10.3390/microorganisms13030493/s1, Data S1: Temperatures; Data S2: Hydrogen peroxide; Data S3: Pathway for pyomelanin synthesis.
