# Supplementary material for: Phenotypic and Molecular Characterization of Pyomelanin-Producing Acinetobacter baumannii ST2Pas;ST1816/ST195Oxf Causing the First European Nosocomial Outbreak
Source: Microorganisms. 2025 Feb 22;13(3):493. doi: 10.3390/microorganisms13030493 (PMC11945678; doi:10.3390/microorganisms13030493)
Supplement: Supplementary file 1 [file microorganisms-13-00493-s001.zip › microorganisms-3385555-supplementary.pdf]

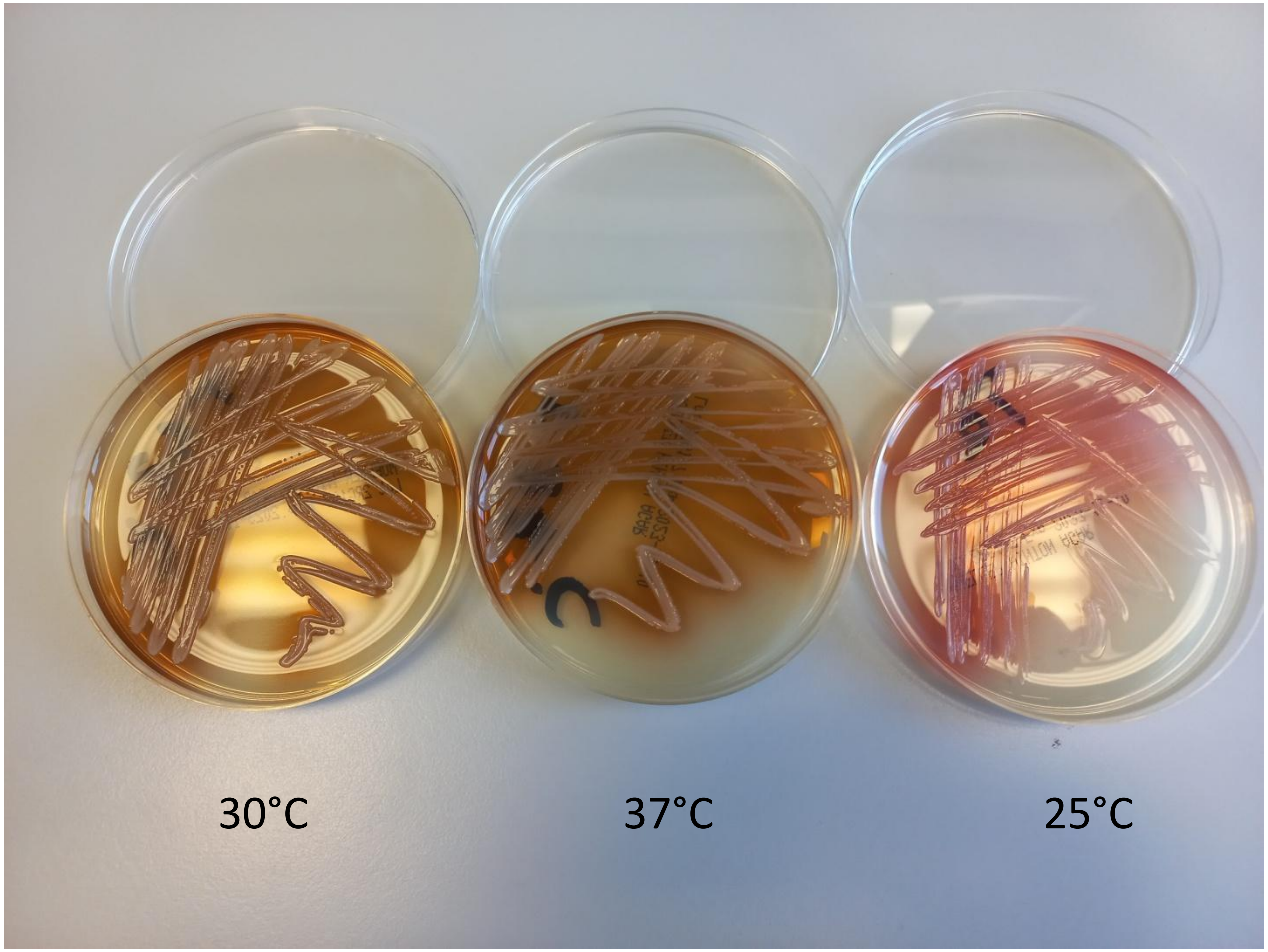

Supplementary Data S1. The production of pyomelanin is more evident at 30°C and 37°C rather than 25°C, after 24h of incubation

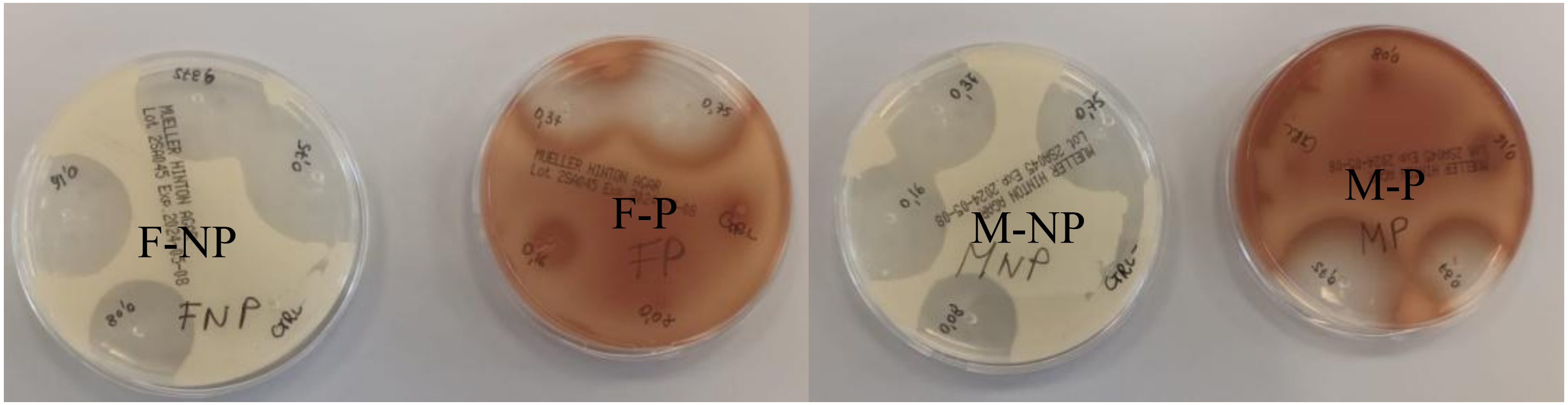

Supplementary Data S2. Two couples of pigmented (M-P, F-P) and not pigmented (M-NP, F-NP) *A. baumannii* strains from the same patients (M and P: each of them had a pigmented and a non pigmented strain) were tested. The pigmented *A. baumannii* strains, MP and FP, showed full growth at 0.08 % of hydrogen peroxide (no inhibition halo was detected), whereas 24 mm of growth inhibition halos were detected for the not pigmented strains at the same hydrogen peroxide concentration.

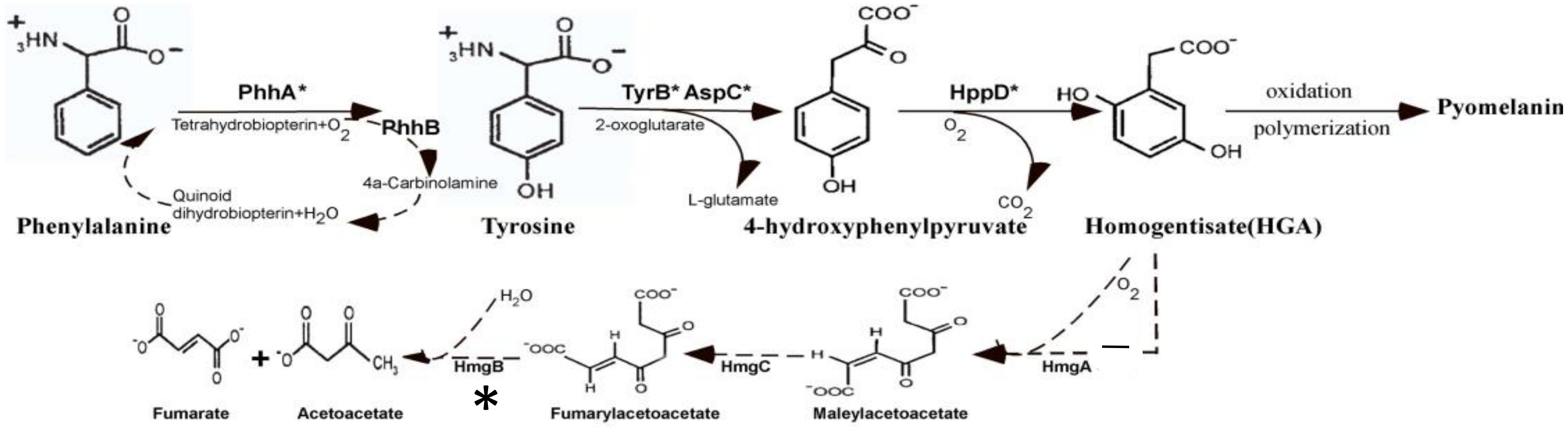

Supplementary Data S3.

Pathway for pyomelanin synthesis and phenylalanine/tyrosine catabolism (modified from https://doi.org/10.1371/journal.pone.0120923**).**

The intermediates of the pathway are indicated. PhhA, phenylalanine hydroxylase; PhhB, 4a-carbinolamine dehydratase; AspC, aromatic amino acid aminotransferase; TyrB, aromatic amino acid aminotransferase; HppD, 4-hydroxyphenylpyruvate dioxygenase; HmgA, homogentisate dioxygenase; HmgB, fumarylacetoacetate hydrolase; HmgC, maleylacetoacetate isomerase.

The asterisk (*) indicates the location of the related genes mutated in this study.
